# Positive surgical margins and biochemical recurrence following minimally-invasive radical prostatectomy – An analysis of outcomes from a UK tertiary referral centre

**DOI:** 10.1186/s12894-017-0262-y

**Published:** 2017-10-02

**Authors:** Ashwin Sachdeva, Rajan Veeratterapillay, Antonia Voysey, Katherine Kelly, Mark I. Johnson, Jonathan Aning, Naeem A. Soomro

**Affiliations:** 1 Department of Urology, Freeman Hospital, Newcastle-upon-Tyne Hospitals NHS Foundation Trust, Newcastle-upon-Tyne, UK; 2 Northern Institute for Cancer Research, Newcastle University, Newcastle-upon-Tyne, UK

## Abstract

**Background:**

Positive surgical margins are a strong prognostic marker of disease outcome following radical prostatectomy, though prior evidence is largely from a PSA-screened population. We therefore aim to evaluate the biochemical recurrence in men with positive surgical margins (PSM) after minimally-invasive radical prostatectomy (MIRP) in a UK tertiary centre.

**Methods:**

Retrospective study of men undergoing laparoscopic or robotic-assisted radical prostatectomy between 2002 and 2014. Men with positive surgical margins (PSM) were identified and their biochemical recurrence (BCR) rate compared with men without PSM. The primary outcome measures were BCR rates and time to BCR. Cox regression was used to estimate adjusted hazard ratios for biochemical recurrence rate (BCR), accounting for potential confounders.

**Results:**

Five hundred ninety-two men were included for analysis. Pre-operative D’Amico risk stratification showed 37.5%, 53.3% and 9.3% of patients in the low, intermediate and high-risk groups, respectively. On final pathological analysis, the proportion of patients with local staging pT2, pT3a and pT3b was 68.8%, 25.2% and 6.1% respectively. Overall positive margin rate was 30.6%. On multivariate analysis, the only pre-operative factor associated with PSM was age >65years. Patients with PSM were more likely to have higher tumour volume and more advanced pathological local stage. The BCR rate was 10.7% in margin-positive patients and 5.1% in margin-negative patients, at median 4.4-year follow-up. Upon multivariate analysis, high pre-operative PSA and high Gleason group were the only significant predictors of BCR (P<0.05).

**Conclusions:**

In comparison to patients with negative surgical margins, those with PSM do not translate into worse medium-term oncological outcomes in the majority of cases amongst our cohort. We found that high pre-operative PSA and high Gleason group were the only significant predictors of BCR.

**Electronic supplementary material:**

The online version of this article (10.1186/s12894-017-0262-y) contains supplementary material, which is available to authorized users.

## Background

Surgical margin status at pathological analysis after radical prostatectomy for prostate cancer is a key metric to define the oncological adequacy of prostate resection [1]. With active surveillance considered the primary management option for low-risk prostate cancer, radical prostatectomy (RP) is increasingly being used for intermediate or high-risk patients, as part of multi-modal therapy [2]. Positive surgical margin (PSM) rates have been associated with RP for higher-risk disease with reported incidence between 11-48% [1–3]. PSM has been highlighted as a risk factor for disease progression after surgery and, as such, margin status has been incorporated as a component of multiple prostate cancer outcome prediction models [4–6].

In contemporary practice, patients with the finding of PSM alone at RP can be managed with PSA surveillance, adjuvant or salvage radiotherapy [7] or entered into clinical trials (for example RADICALS) [1, 4]. For patients with biochemical relapse (BCR) following RP, the European Association of Urology advises the use of salvage radiotherapy with at least 66Gy at a PSA level of < 0.5ng/ml [8].

In a recent report regarding contemporary MIRP in the UK, the authors reported a trend towards increasing use of MIRP amongst patients with high risk disease, but with a high positive surgical margin rate of 33.6% amongst patients with pT3 disease [9]. However, longer-term oncological outcome data following MIRP in the UK are lacking. Furthermore, only a small percentage of patients in the UK are diagnosed with prostate cancer by PSA screening [10], in comparison to the widespread adoption of routine PSA screening in USA and some European countries [11]. Therefore, oncological outcomes of RP in a relatively unscreened UK population may be hypothesised to vary from those previously reported for screened cohorts.

In this report we describe the PSM rates in a consecutive series of patients undergoing minimally invasive radical prostatectomy at a large UK tertiary referral centre. We explore the factors predicting PSM and assess the medium term oncological outcomes as regards to biochemical recurrence rates.

## Methods

### Study population

Following approval by the local audit and research department, consecutive patients undergoing minimally invasive radical prostatectomy (MIRP - laparoscopic or robotic assisted) at our institution (between January 2002 and April 2014) were identified from a departmental database. Excised specimens underwent centralised pathological review. Patients undergoing open radical prostatectomy during this period were excluded. Retrospective review of their medical electronic records, histopathology data and biochemistry investigations was conducted. Data extracted included preoperative parameters (demographics, PSA, prostate biopsy Gleason score, clinical stage), operative details (technique and whether lymphadenectomy was performed) and postoperative radical prostatectomy pathology (presence of positive surgical, tumour Gleason score, tumour stage, tumour volume). Patients who had detectable PSA (ie >0.1ng/mol) 12 weeks post-operatively, less than 12 months of follow-up or those for whom PSA follow-up data were unavailable, were excluded from this study.

### Follow-up and biochemical recurrence

PSA follow-up data were captured. BCR was defined as undetectable PSA post radical prostatectomy, which subsequently rose to ≥ 0.2ng/mol. For patients with biochemical recurrence, the need and timing of adjuvant therapy (if any) was recorded.

### Statistical analysis

Patient and disease characteristics between patients stratified by incidence of surgical margins and biochemical recurrence were compared using the Chi square test. Student’s T test was used to compare means and Mann-Whitney U test was used to compare medians. Potential co-variates including age, pre-operative PSA, Gleason score, and tumour volume, were included in a Cox regression model to estimate adjusted hazard ratios for positive surgical margins and biochemical recurrence. Kaplan-Meier survival curves were built using the time of biochemical recurrence as a failure event. A false discovery rate adjustment was applied for multiple comparisons. Results were deemed to be statistically significant if p value was less than 0.05.

## Results

### The overall positive surgical margin rate was 30.6%

In total 592 men underwent minimally invasive radical prostatectomy (MIRP) between 2002 and 2012. Median age at MIRP was 63 years (IQR 58-67 years). Median PSA at diagnosis was 7.9ng/ml (IQR 5.7-11.8ng/ml) and median follow-up was 52.8 months (IQR 25.0-73.6 months). Demographic data is summarised in Table 1. Pre-operative D’Amico risk stratification noted 37.5% of patients in the low-risk group, 53.3% in the intermediate-risk group and 9.3% in the high-risk group. 393 patients underwent laparoscopic RP (LRP) and 199 underwent robotic-assisted RP (RARP). On final pathological analysis, the proportion of patients with local staging pT2, pT3a and pT3b was 68.8%, 25.2% and 6.1% respectively. Lymph node sampling was performed on 41.7% patients (247 of 592), of whom 19 patients (8.0%) had evidence of lymph node involvement. Positive surgical margins (PSM) were identified in 181/592 (30.6%) excised pathological specimens.

**Table 1:** Patient & disease characteristics overall, stratified by surgical margin status

Variable	Patients with positive margins	Patients with negative margins	Overall	*p* value
N (%)	181 (30.6)	411 (69.4)	592	
Patient age at time of surgery				
Under 65 years	82 (45.3)	252 (61.3)	334 (56.4)	0.009
65 years and above	99 (54.7)	159 (38.7)	258 (43.6)	
Pre-op PSA				
0.0-9.9	103 (57.9)	276 (68.3)	379 (65.1)	0.03
10.0-19.9	58 (32.6)	110 (27.2)	168 (28.9)	
20.0 and above	17 (9.6)	19 (4.5)	35 (6.0)	
Missing	2	7	9	
Gleason score on biopsy				
Group 1 (GS 2-6)	81 (44.8)	191 (46.5)	272 (46.0)	0.5
Group 2 (GS 3+4)	74 (40.9)	165 (40.2)	239 (40.4)	
Group 3 (GS 4+3)	20 (11.0)	35 (8.5)	55 (9.3)	
Group 4 (GS 8)	3 (1.7)	16 (3.9)	19 (3.2)	
Group 5 (GS 9-10)	3 (1.7)	4 (1.0)	7 (1.2)	
Pathological Gleason score				
Group 1 (GS 2-6)	26 (14.4)	117 (28.5)	143 (24.2)	<0.001
Group 2 (GS 3+4)	92 (50.8)	218 (54.0)	310 (52.4)	
Group 3 (GS 4+3)	33 (18.2)	40 (9.7)	73 (12.3)	
Group 4 (GS 8)	20 (11.1)	31 (7.5)	51 (8.6)	
Group 5 (GS 9-10)	10 (5.5)	5 (1.2)	15 (2.5)	
Pathological tumour stage				
pT2	89 (49.2)	318 (77.4)	407 (68.8)	<0.001
pT3a	68 (37.6)	81 (19.7)	149 (25.2)	
pT3b	24 (13.3)	12 (2.9)	36 (6.1)	
Lymph node involvement	10 (11.8)	7 (4.6)	17 (7.2)	0.06
Tumour volume of excised specimen				
Median (IQR)	3.9 (1.8-6.7)	2.02 (0.81-4.0)	2.4 (1.0-5.1)	<0.001
Surgical approach				
LRP	109 (60.2)	284 (69.1)	393 (66.4)	0.05
RARP	72 (39.8)	157 (30.9)	199 (33.6)	
Post-operative PSA*				
Biochemical recurrence (%)	15/140 (10.7)	18 of 350 (5.1)	33 of 490 (6.7)	0.045
Median time to BCR (in months, IQR)*	10.9 (3.7-24.5)	13.1 (4.9-37.7)	12.1 (4.9-31.4)	0.5

*PSA data available for 532 of 592 patients. 490 patients had undetectable PSA post-operatively

### PSM was associated with older age but not biopsy Gleason score

On univariate analysis (Table 1), the only preoperative factors associated with PSM were patient age >65years and PSA >10ng/ml. Patients with PSM were also more likely to have higher Gleason score on final pathology, higher tumour volume and more advanced pathological local stage. The overall PSM was marginally higher following RARP, as compared to LRP (36.2% vs 27.7%, *p* = 0.05). There was no significant change in PSM over time (LRP: *p* = 0.5; RARP: *p* = 0.4).

On multivariate logistic regression (Table 2), key factors predictive of PSM were patient age >65 years (OR 2.11, 95% CI 1.41-3.15), higher pT stage (pT3a OR 2.69, 95% CI 1.73-4.18; pT3b OR 6.35, 95% CI 2.77-14.57), larger tumour volume (OR 1.09, 95% CI 1.03-1.16), and the use of a robotic-approach (OR 2.52, 95% CI 1.18-5.37).

**Table 2:** Logistic regression results with 95% CI & p values (what factors predict PSM)

	Univariate analysis			Multivariate analysis		
Variable	OR	95% CI	*p* value	OR	95% CI	*p* value
Year of procedure						
2002-2009	1.00	-	-	1.00	-	-
2010-2012	1.15	0.76-1.73	0.5	0.72	0.43-1.19	0.2
2013-2014	1.25	0.79-1.96	0.4	0.37	0.15-0.93	0.03
Patient age at time of surgery						
Under 65 years	1.00	-	-	1.00	-	-
65 years & above	1.89	1.33-2.70	<0.001	2.11	1.41-3.15	<0.001
Pre-op PSA						
0.0-9.9	1.00	-	-	1.00	-	-
10.0-19.9	1.41	0.96-2.09	0.2	1.05	0.67-1.65	0.8
20.0 and above	2.53	1.26-5.10	0.02	1.30	0.56-3.00	0.5
Gleason score on biopsy						
Group 1 (GS 2-6)	1.00	-	-	1.00	-	-
Group 2 (GS 3+4)	1.06	0.72-1.54	0.8	0.75	0.48-1.17	0.2
Group 3 (GS 4+3)	1.35	0.73-2.47	0.4	0.86	0.43-1.69	0.6
Group 4 (GS 8)	0.44	0.13-1.56	0.3	0.22	0.05-0.90	0.04
Group 5 (GS 9-10)	1.77	0.39-8.08	0.6	0.86	0.15-4.89	0.9
Pathological tumour stage						
pT2	1.00	-	-	1.00	-	-
pT3a	3.00	2.01-4.47	<0.001	2.69	1.73-4.18	<0.001
pT3b	7.15	3.44-14.85	<0.001	6.35	2.77-14.57	<0.001
Median tumour volume of excised specimen	1.16	1.10-1.22	<0.001	1.09	1.03-1.16	0.005
Surgical modality						
LRP	1.00	-	-	1.00	-	-
RARP	1.48	1.03-2.13	0.08	2.52	1.18-5.37	0.02

### The biochemical recurrence rate in PSM was 10.7%

**Fig. 1:**
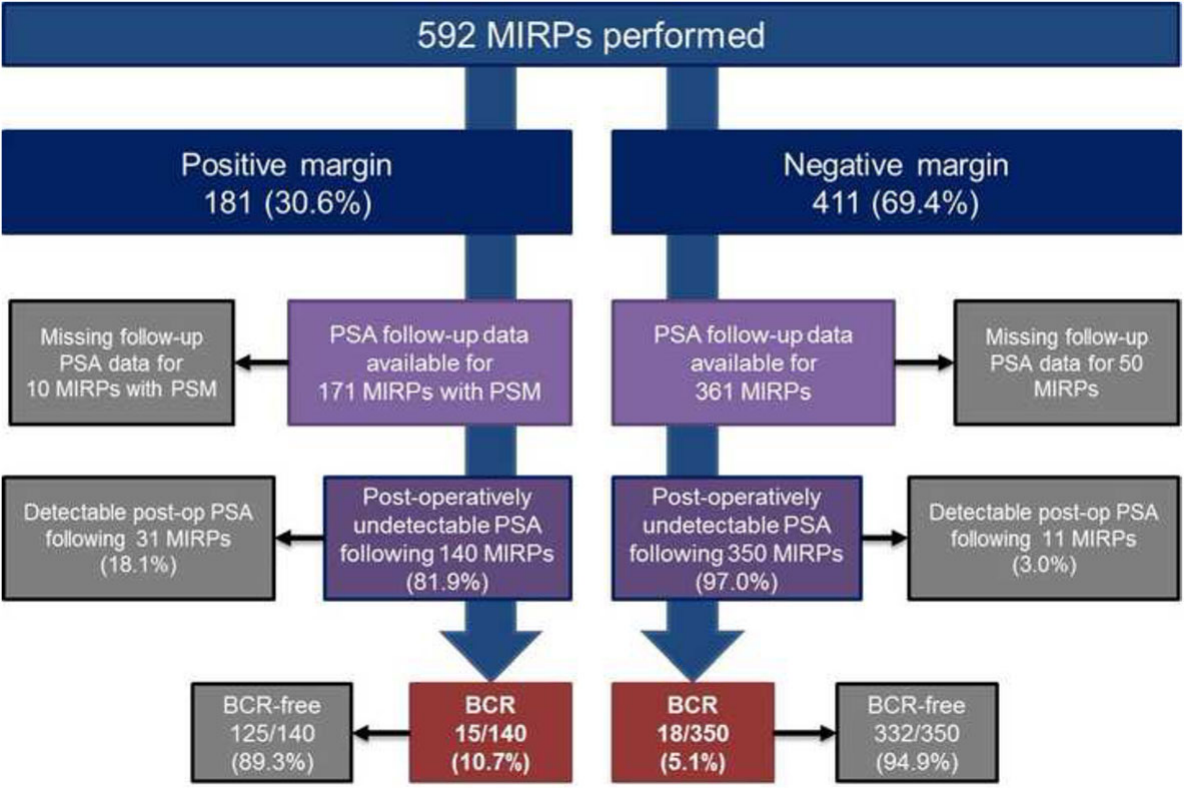
Flow diagram of study population

PSA follow up data were available for 532 patients (89.9% of the cohort, Fig. 1) at a median follow-up of 30.3 months (16.0 - 52.7). Of these, 42 patients had detectable PSA post-operatively and were therefore excluded. In comparison to patients with undetectable PSA, these 42 patients tended to have more aggressive disease (Additional file 1: Table S1).

**Fig. 2:**
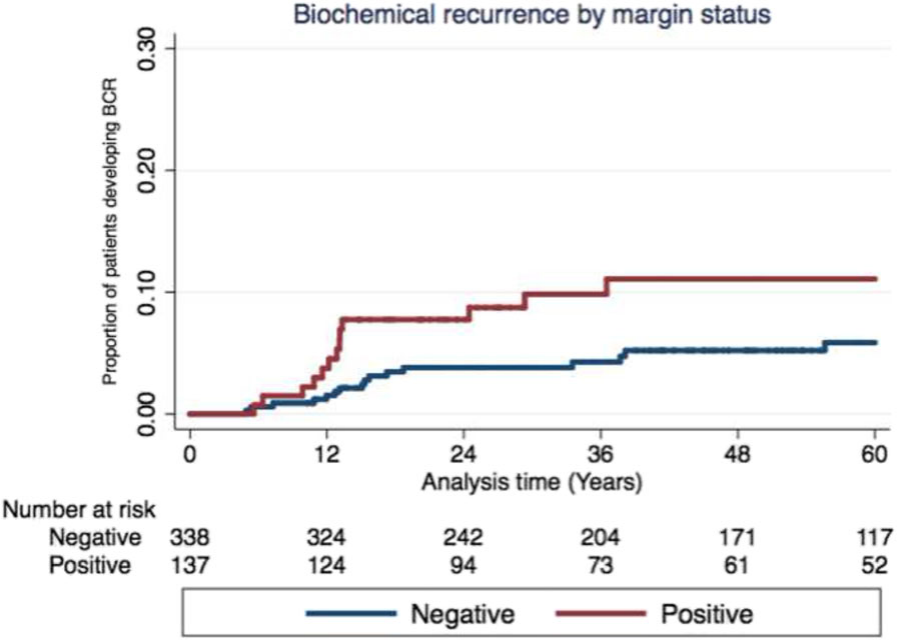
Kaplan Meier survival analysis of time to biochemical recurrence stratified by margin status

The overall biochemical recurrence rate (BCR) was 6.7% with a median time to BCR of 13.3 (11.6-33.5) months. Comparing PSM and NSM, the BCR at median follow-up of 52.8 months (IQR 25.0-73.6 months) was 10.7% and 5.1% respectively (p 0.026, Fig. 2). There was no difference in time to BCR between patients with PSM and NSM with a median time to BCR of 13.1 (10.8-29.3) months and 15.5 (12.0-37.7) respectively (*p* 0.49).

Upon univariate analysis, pre-operative PSA greater than 10ng/ml, larger tumour volume and PSM were associated with BCR (Table 3). However, upon multivariate Cox regression analysis to adjust for potential confounders, higher pre-operative PSA and higher Gleason group disease were associated with BCR (Additional file 1: Table S2). Of note, margin status was not associated with development of BCR.

**Table 3:** Patient & disease characteristics of patients with PSM, stratified by biochemical recurrence at end of study period

Variable	BCR-free	BCR	All PSMs	*p* value
N (%)	125 (89.3)	15 (10.7)	140	
Patient age at time of surgery				
Under 65 years	60 (48.0)	7 (46.7)	67 (47.9)	0.4
65 years and above	65 (52.0)	8 (53.3)	73 (52.1)	
Pre-op PSA				
0.0-9.9	81 (64.8)	4 (26.7)	85 (60.7)	0.003
10.0-19.9	38 (30.4)	7 (46.7)	45 (32.1)	
20.0 and above	6 (4.8)	4 (26.7)	10 (7.2)	
Pathological Gleason score				
Group 1 (GS 2-6)	20 (16.0)	0	20 (14.3)	0.4
Group 2 (GS 3+4)	59 (55.2)	8 (53.3)	77 (55.0)	
Group 3 (GS 4+3)	18 (14.4)	5 (33.3)	23 (16.4)	
Group 4 (GS 8)	13 (10.4)	2 (13.3)	15 (10.7)	
Group 5 (GS 9-10)	5 (4.0)	0	5 (3.6)	
Surgical approach				
LRP	75 (60.0)	10 (66.7)	85 (60.7)	0.6
RARP	50 (40.0)	5 (33.3)	55 (39.3)	
Tumour volume of excised specimen				
Median (IQR)	3.7 (1.7-6.6)	5.1 (2.5-10.2)	3.8 (1.8-6.7)	<0.001
Pathological tumour stage				
pT2	67 (53.6)	5 (33.3)	72 (51.4)	0.4
pT3a	44 (35.2)	6 (40.0)	50 (35.7)	
pT3b	14 (11.2)	4 (26.7)	18 (12.9)	
Lymph node involvement	7 (13.5)	0 (0.0)	0 (11.5)	0.4
Margin location				
Apex	33 (26.4)	4 (26.7)	37 (26.4)	0.4
Base	23 (18.4)	1 (6.7)	24 (17.1)	
Multifocal	19 (15.2)	5 (33.3)	24 (17.1)	
Other	50 (40.0)	5 (33.3)	55 (39.3)	
Adjuvant therapy				
Salvage radiotherapy	12 of 41 (29.3)	10 of 14 (64.1)	22 of 55 (40.0)	
Radiotherapy & hormones	0	1 of 14 (7.2)	1 of 55 (1.8)	
Missing	84	1	85	

Note: Patients with detectable PSA or missing PSA data post-operatively were excluded

## Discussion

This is the largest reported single-centre UK series of oncological outcomes, including biochemical recurrence rates, following MIRP. The overall PSM in this cohort of 592 patients was 30.6%. Upon adjustment for potential confounders, the only variables associated with greater incidence of PSM were older age at time of surgery, pT3 stage disease, higher tumour volume, and use of a RARP approach.

Comparative data indicate that the incidence of positive margins is equivalent among the open, laparoscopic, and RARP approaches [12]. A recent meta-analysis reported a 15% mean rate of PSMs in RARP series published between 2008 and 2011, with a range of 6.5–32% [3]. PSM rates in contemporary series range between 10.4 to 31.1%. The largest report, by Wright et al, was based on a database study of 65,633 patients having radical prostatectomy. PSMs were reported in 21.2% of cases and were more common in pT3a than pT2 tumours (44% vs 18%, p <0.001) and higher grade tumours (28% vs 18%, p <0.001) [13]. Reported RARP series have noted a PSM rate of 15.7-29.5% [9, 14–18]. Preoperative PSA, prostate volume on trans-rectal ultrasound, clinical T (cT) stage, and pathological stage (pT2 vs pT3) have been reported as independent predictors of the presence of any PSM, while cT stage and biopsy Gleason score have been reported as predictors of posterolateral PSM [14, 15].

From a UK perspective, of all 2,163 radical prostatectomies (54.6% laparoscopic and 19.6% RARP) entered in the British Association of Urological Surgeons database in 2011, the overall PSM was 42.3% with no difference between laparoscopic (26.6%) and robotic (22.5%) cases [19]. Our findings of PSM rates are comparable to published reports on minimally invasive prostatectomy. We do note however a higher PSM rate in patients undergoing robotic surgery (36.2%) in our cohort and this may be representative of a learning curve effect. It is worth noting an increasing proportion of patients being offered surgery for high-risk disease. A recent study of robotic RP in this group was reported by Kang et al who reported a 25.1% PSM rate, and that higher tumour stage and volume were associated with PSM [20]. Furthermore, a recent UK series reported that accumulated experience with robotic RP was associated with a temporal decrease in PSM rate (22.5% in 2005-2008 vs 19.8% in 2013-15), despite an increase in the proportion of patients having surgery for higher risk disease [9].

In our study, patients with PSM were more likely to develop biochemical recurrence (10.7%) versus those with negative margins (5.1%). The median time to BCR for patients with PSM was 13.1 months. On multivariate analysis, factors predicting biochemical failure were high pre-operative PSA and higher pathological Gleason group at MIRP. The oncological implications of a positive surgical margin at radical prostatectomy are difficult to predict [1]. Nine large contemporary studies have investigated the impact of PSMs on biochemical recurrence rates, metastatic progression and prostate-cancer mortality (Table 4). Whilst all studies found PSMs to be associated with a higher risk of BCR, data on time to metastatic progression and death were less clear. Increased risk of PCa death was noted in men with positive compared with negative surgical margins, at 4.2 year [13] and 10 year follow-up [21]. However, this impact was fairly marginal relative to the impact of Gleason score and tumour stage on pathological assessment of the excised prostate [21]. From the literature, it is apparent that PSM increases the risk of disease recurrence but the range of risk and the time to event (death from prostate cancer) are very wide, depending mostly on the presence or absence of other risk modifiers. Even if the risk is real, competing causes of mortality may obscure the predictive value of PSMs for death due to PCa [1].

**Table 4:** Comparison of PSM & BCR with previously published data

		Ref	Study period	Total number of patients	Pre-operative PSA mean (median) in ng/mL	Median tumour vol. (cc)	Surgical approach			PSM rate (%)	Stratified by pathological stage			% BCR	Stratified by margin status		Median follow-up
							% LRP	% Open	% RARP		pT2	pT3a	pT3b		PSM	NSM	
This study	Freeman Hospital, UK	-	2002-2014	592	3.65 (2.4)	PSM 3.9 NSM 2.0	66.4%	-	33.6%	30.6%	21.9%	45.6%	66.7%	6.7%	10.7%	5.1%	4.4 years
Wright et al.	SEER, USA	[13]	1998-2006	65,633	-	-	-	-	-	21.2%	17.7%	43.8%	-	-	-	-	N/A
Mauermann et al.	Quebec, Canada	[22]	1987-2010	1,712	8.5 (6.8)	-	10.6%	89.4%	-	34.5%	27.6%	54.1%	49.5%*	16.4%	26.7%	10.9%	6 years
Ficarra et al.	Padua, Italy	[14]	2005-2008	322	-	-	-	-	100.0%	29.5%	10.6%	57.5%	72.2%	3.2%	6.2%	1.8%	1 year
Patel et al.	Multi-institution	[15]	2002-2009	8,418	-	-	-	-	100.0%	15.7%	9.5%	33.2%	48.2%	-	-	-	N/A
Boorjian et al.	Mayo Clinic, USA	[32]	1990-2006	11,729**	PSM (5.9) NSM (8.1)	PSM 3.3 NSM 1.1	-	100.0%	-	31.1%	23.4%	52.9%	55.4%	25.6%	44.0%	23.0%	8.2 years
Chalfin et al.	John Hopkins, USA	[21]	1982-2011	4,461	6.8 (5.4)	-	-	100.0%	-	10.4%	14.6%	22.2%	31.5%	16.7%	54.5%	12.3%	10 years
Sooriakumaran et al.	Stockholm	[16]	2002-2006	944	6.4	-	-	-	100.0%	21.6%	16.0%	33.3%	57.9%	15.2%	29.0%	12.0%	6.3 years
Evans et al.	Victoria, Australia	[33]	2008-2012	2219	-	-	8.1%	53.0%	38.8%	27.2%	16.1%	50.7%		-	-	-	N/A
Sukumar et al.	Detroit, USA	[17]	2001-2010	4803	6.1	Mean 7.5	-	-	100.0%	24.7%	11.2%	50.5%		9.8%	-	-	2.2 years
Abdollah et al.	Multi-institution	[18]	2001-2010	5670	6.4 (5.2)	-			100.0%	24.0%		34.3%***		14.1%	-	-	4.2 years
Gnanapragasam et al.	Cambridge, UK	[9]	2005-2015	1500	8.5 (7.3)	-			100.0%	21.5%	9.1%	29.6%	53.6%	-	-	-	N/A
Range										10.4-34.5%	9.1-27.6%	22.2-57.4%	31.5-72.2%	3.2-25.6%	6.2-54.5%	1.8-23.0%	1-10 years

*Includes pT4 (2002 TNM staging, changed to pT3b in 2010); **Patients with N1 disease excluded (PSM rate 295/484 = 61%); ***High-risk D'Amico group

Biochemical recurrence is a marker of disease progression and associated with poor prognosis. Previous reports of BCR in patients with PSM are variable due to differing lengths of follow-up and surgical approach. Studies with patients predominantly undergoing open RP have reported a BCR rate of 26.7% at 6 years [22] to 54.3% at 10 years [21]. In contrast, shorter follow-up data is available for patients with PSM following MIRP, with a BCR rate ranging from 6.2% at 1 year [14] to 29.0% at 6.3 years [23]. A recent multi-institutional study reported outcomes of over 5000 patients following RARP and reported BCR rates of 14.1% at a median follow-up of 4.2 years [18]. Our results are within this range and, based upon previous data, it is likely that additional patients may go on to develop BCR at longer follow-up.

The choice of therapeutic strategy for patients with PSM remains controversial. Recent evidence suggests that adjuvant radiotherapy may lead to a 50–60% reduction in the risk of PSA progression in men with pathologically advanced prostate cancer [24, 25]. However, not all men with PSMs are destined to have treatment failure and indeed the majority of men with isolated PSMs, with or without extra-prostatic extension, are cured after RP alone [1, 3]. Therefore, recommending adjuvant radiotherapy to all men with isolated PSMs should be done with caution especially when factoring in the added morbidity associated with radiotherapy treatment.

Detectable PSA following radical prostatectomy is often considered treatment failure. In our cohort, 7.9% had detectable PSA post-operatively, and were excluded from subsequent analyses. These patients tended to have more aggressive disease and were more likely to have had positive surgical margins (73.8% in patients with detectable PSA, vs 28.6% in patients with undetectable PSA post-operatively, p<0.001, Additional file 1: Table S1). Similarly, Koulikov et al reported similar findings, whereby patients with low-detectable PSA (>0.03 and <0.2ng/ml) and PSA velocity >0.05ng/year, were more likely to have positive surgical margins and an increased incidence of biochemical recurrence [26]. These data suggest that men with low-detectable PSA post-prostatectomy may be divided into two groups based upon PSA velocity, those with stable PSA who do not often develop biochemical recurrence and those with unstable PSA who go on to develop biochemical recurrence. There is some evidence to suggest that this subgroup of patients may benefit from adjuvant radiotherapy [27].

There are some limitations to this study due to retrospective design and lack of PSA follow-up data for all patients. This is due to patients moving to their local units for follow-up. While previous reports and ours have predominantly focussed on reviewing the influence of pre-operative and surgical factors on long-term outcome, it is prudent to also note the potential impact of pathological features at the tumour margin. Pathological data regarding length of positive margin and grade pattern at the margin have previously been reported to predict BCR [28]. However, such data were not available for inclusion in our study. Moreover, molecular and biochemical features at tumour margins [29, 30] may also impact long term oncological outcomes, and subtyping positive margins based upon such features may provide a more effective method of identifying patients with PSM likely to benefit from personalised adjuvant therapies, as has been proposed for resection of other malignancies [31]. Lastly, this is a consecutive series of patients with an increasing volume of MIRPs performed each year. Therefore, it is difficult to accurately control for the effect of the learning curve. However, we adjusted for year of procedure, which may help adjust for this potential confounder to some degree. Interestingly, our data compares well to other series though we await the long-term maturation of this cohort.

## Conclusion

In comparison to patients with negative surgical margins, those with PSM do not translate into worse medium-term oncological outcomes in the majority of cases amongst our cohort. We found that high pre-operative PSA and Gleason grade group were the only robust predictors of BCR.

## Additional file

Additional file 1: Table S1. Patient & disease characteristics overall, stratified by post-operative PSA status (detectable vs undetectable). **Table S2.** Multivariate evaluation of factors predicting BCR at 5-year follow-up using Cox regression. Results are reported as adjusted hazard ratios, with 95% confidence intervals. (DOCX 20 kb)

## Abbreviations

BCR: Biochemical recurrence
LRP: Laparoscopic radical prostatectomy
MIRP: Minimally-invasive radical prostatectomy
NSM: Negative surgical margin
PSA: Prostate specific antigen
PSM: Positive surgical margin
RARP: Robotic-assisted radical prostatectomy
