# Supplementary material for: Positive surgical margins and biochemical recurrence following minimally-invasive radical prostatectomy – An analysis of outcomes from a UK tertiary referral centre
Source: BMC Urol. 2017 Oct 2;17:91. doi: 10.1186/s12894-017-0262-y (PMC5625596; doi:10.1186/s12894-017-0262-y)
Supplement: Additional file 1: Table S1. — Patient & disease characteristics overall, stratified by post-operative PSA status (detectable vs undetectable). Table S2. Multivariate evaluation of factors predicting BCR at 5-year follow-up using Cox regression. Results are reported as adjusted hazard ratios, with 95% confidence intervals. (DOCX 20 kb) [file 12894_2017_262_MOESM1_ESM.docx]

**Supplementary data**

**Table S1:** Patient & disease characteristics overall, stratified by post-operative PSA status (detectable vs undetectable).

| **Variable** | **Patients with detectable PSA post-operatively** | **Patients with undetectable PSA post-operatively** | **FDR-adjusted p value** |
| --- | --- | --- | --- |
| **N (%)** | 42 (7.9%) | 490 (92.1%) |  |
| **Patient age at time of surgery** |  |  |  |
| Under 65 years | 11 (26.2) | 277 (56.5) | 0.002 |
| 65 years and above | 31 (73.8) | 213 (43.5) |  |
| **Pre-operative PSA** |  |  |  |
| 0.0-9.9 | 17 (40.5) | 316 (65.1) | <0.001 |
| 10.0-19.9 | 18 (42.9) | 143 (29.5) |  |
| 20.0 and above | 7 (16.7) | 26 (5.4) |  |
| *Missing* | - | 5 |  |
| **Gleason score at biopsy** |  |  |  |
| Group 1 (GS 2-6) | 13 (31.0) | 232 (47.4) | 0.01 |
| Group 2 (GS 3+4) | 17 (40.5) | 198 (40.4) |  |
| Group 3 (GS 4+3) | 8 (19.1) | 43 (8.8) |  |
| Group 4 (GS 8) | 2 (4.8) | 14 (2.9) |  |
| Group 5 (GS 9-10) | 2 (4.8) | 3 (0.6) |  |
| **Pathological Gleason score** |  |  |  |
| Group 1 (GS 2-6) | 4 (9.5) | 119 (24.3) | 0.005 |
| Group 2 (GS 3+4) | 19 (45.2) | 263 (53.7) |  |
| Group 3 (GS 4+3) | 9 (21.4) | 59 (12.0) |  |
| Group 4 (GS 8) | 6 (14.3) | 39 (8.0) |  |
| Group 5 (GS 9-10) | 4 (9.5) | 10 (2.0) |  |
| **Tumour volume of excised specimen** |  |  |  |
| Median (IQR) | 3.86 (1.84-6.24) | 2.4 (1.00-5.07) | 0.015 |
| **Pathological tumour stage** |  |  |  |
| pT2 | 19 (45.2) | 342 (69.8) | 0.005 |
| pT3a | 18 (42.9) | 118 (24.1) |  |
| pT3b | 5 (11.9) | 30 (6.1) |  |
| *Lymph node involvement* | *4 (14.8)* | *14 (7.2)* | *0.195* |
| **Surgical approach** |  |  |  |
| LRP | 25 (59.5) | 317 (64.7) | 0.502 |
| RARP | 17 (40.5) | 173 (35.3) |  |
| **Margin status** |  |  |  |
| Positive surgical margin | 31 (73.8) | 140 (28.6) | <0.001 |

**Table S2:** Multivariate evaluation of factors predicting BCR at 5-year follow-up using Cox regression. Results are reported as adjusted hazard ratios, with 95% confidence intervals.

|  | **Univariate analysis** | | | **Multivariate analysis** | | |
| --- | --- | --- | --- | --- | --- | --- |
| **Variable** | **Adj HR** | **95% CI** | **p value** | **Adj HR** | **95% CI** | **p value** |
| **Patient age at time** | 1.03 | 0.97-1.10 | 0.347 | 1.00 | 0.94-1.07 | 0.917 |
|  |  |  |  |  |  |  |
| **Pre-op PSA** | 1.10 | 1.05-1.14 | <0.001 | 1.06 | 1.01-1.11 | 0.017 |
|  |  |  |  |  |  |  |
| **Pathological Gleason score** |  |  |  |  |  |  |
| Group 1 (GS 2-6) | 1.00 | - | - | 1.00 | - | - |
| Group 2 (GS 3+4) | 6.78 | 0.89-51.99 | 0.065 | 4.93 | 0.63-38.31 | 0.127 |
| Group 3 (GS 4+3) | 24.02 | 3.03-190.21 | 0.003 | 13.52 | 1.60-114.08 | 0.017 |
| Group 4 (GS 8) | 21.84 | 2.55-187.30 | 0.005 | 11.39 | 1.24-104.34 | 0.031 |
| Group 5 (GS 9-10) | 24.27 | 1.50-392.03 | 0.025 | 18.84 | 1.10-323.06 | 0.043 |
|  |  |  |  |  |  |  |
| **Pathological tumour stage** |  |  |  |  |  |  |
| pT2 | 1.00 | - | - | 1.00 | - | - |
| pT3a | 3.01 | 1.35-6.71 | 0.007 | 1.81 | 0.77-4.21 | 0.171 |
| pT3b | 5.45 | 1.92-15.48 | 0.001 | 1.70 | 0.47-6.17 | 0.417 |
|  |  |  |  |  |  |  |
| **Tumour volume of excised specimen** | 1.07 | 1.02-1.11 | 0.004 | 1.02 | 0.94-1.09 | 0.671 |
|  |  |  |  |  |  |  |
| **Margin status** |  |  |  |  |  |  |
| Negative | 1.00 | - | - | 1.00 | - | - |
| Positive | 2.14 | 1.03-4.46 | 0.041 | 1.23 | 0.55-2.72 | 0.617 |
